# Tocotrienols inhibit lipopolysaccharide-induced pro-inflammatory cytokines in macrophages of female mice

**DOI:** 10.1186/1476-511X-9-143

**Published:** 2010-12-16

**Authors:** Asaf A Qureshi, Julia C Reis, Christopher J Papasian, David C Morrison, Nilofer Qureshi

**Affiliations:** 1 Departments of Basic Medical Sciences, University of Missouri-Kansas City, 2411 Holmes Street, Kansas City, MO 64108, USA; 2 Department of Pharmacology/Toxicology, School of Pharmacy, 2464 Charlotte Street, Kansas City, MO 64108, USA

## Abstract

**Background:**

Inflammation has been implicated in cardiovascular disease, and the important role of proteasomes in the development of inflammation and other macrophage functions has been demonstrated. Tocotrienols are potent hypocholesterolemic agents that inhibit β-hydroxy-β-methylglutaryl coenzyme A reductase activity, which is degraded via the ubiquitin-proteasome pathway. Our objective was to evaluate the effect of tocotrienols in reducing inflammation. Lipopolysaccharide (LPS) was used as a prototype for inflammation in murine RAW 264.7 cells and BALB/c female mice.

**Results:**

The present results clearly demonstrate that α-, γ-, or δ-tocotrienol treatments inhibit the chymotrypsin-like activity of 20 S rabbit muscle proteasomes (> 50%; *P *< 0.05). Chymotrypsin, trypsin, and post-glutamase activities were decreased > 40% (*P *< 0.05) with low concentrations (< 80 μM), and then increased gradually with concentrations of (80 - 640 μM) in RAW 264.7 whole cells. Tocotrienols showed 9 - 33% (*P *< 0.05) inhibitions in TNF-α secretion in LPS-stimulated RAW 264.7 cells. Results of experiments carried out in BALB/c mice demonstrated that serum levels of TNF-α after LPS treatment were also reduced (20 - 48%; *P *< 0.05) by tocotrienols with doses of 1 and 10 μg/kg, and a corresponding rise in serum levels of corticosterone (19 - 41%; *P *< 0.05) and adrenocorticotropic hormone (81 - 145%; *P *< 0.02) was observed at higher concentrations (40 μM). Maximal inhibition of LPS-induced TNF-α was obtained with δ-tocotrienol (10 μg/kg). Low concentrations of δ-Tocotrienols (< 20 μM) blocked LPS-induced gene expression of TNF-α, IL-1β, IL-6 and iNOS (> 40%), while higher concentrations (40 μM) increased gene expression of the latter in peritoneal macrophages (prepared from BALB/c mice) as compared to control group.

**Conclusions:**

These results represent a novel approach by using natural products, such as tocotrienols as proteasome modulators, which may lead to the development of new dietary supplements of tocotrienols for cardiovascular diseases, as well as others that are based on inflammation.

## Background

Lipopolysaccharide (LPS), which is expressed on the outer membrane of essentially all Gram-negative bacteria, is a potent inducer of pro-inflammatory cytokines, including tumor necrosis factor-α (TNF-α interleukin-1β (IL-1β), IL-6, IL-8, arachidonic acid metabolites and nitric oxide [1]. LPS can also induce corticosteroid production by the host, which tends to suppress further production of pro-inflammatory cytokines. Some conditions leading to dysregulated production of inflammatory cytokines by the host can produce profound alterations in metabolic, cardiovascular, immunological, haemostatic, and endocrine functions, which may ultimately lead to septic shock [1-3]. Less profound inflammatory responses have also been implicated in the pathogenesis of atherosclerosis, cancer, stroke and diabetes in human subjects [4-7].

Proteasomes are essential for numerous physiological processes, including signal transduction, transcriptional activation, cell cycle progression, and certain immune cell functions [8]. We have reported a potentially important central role for proteasomes in inflammation and other macrophage functions [8]. Proteasomes often exist as 26 S multi-subunit complexes containing a 20 S proteolytic proteasome and a 19 S regulatory complex. Correspondingly, the 20 S proteasome is comprised of a variety of distinct protein subunits that account for the different proteolytic activities of the 20 S proteasome. Several different exogenous inhibitors or activators of proteasome function have been described, and these inhibitors act by blocking, or activating, the proteolytic activity of the individual protein subunits of the 20 S proteasome.

**Figure 1:**
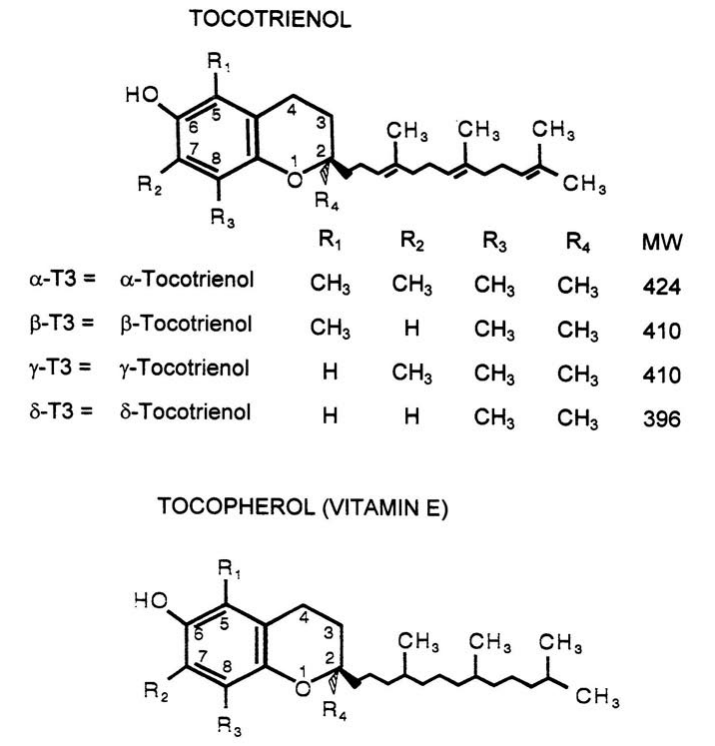
**Chemical structures of various isomers of tocopherols and tocotrienols**.

We, and others, have reported that tocotrienols interfere with the formation of atherosclerotic plaque, and possess hypocholesterolemic, antioxidant, anti-inflammatory, antithrombotic, and anti-proliferative (anticancer) properties [9-22]. Tocotrienols are naturally occurring compounds containing a chroman ring and a farnesylated unsaturated side-chain with analogs of α-, β-, γ- and δ-type. These tocotrienols are minor constituents of natural vitamin E (predominantly α-tocopherol) which has a saturated side-chain attached to a chroman ring (Figure 1). Tocotrienols lower serum total- and LDL-cholesterol levels by inhibiting hepatic β-hydroxy-β-methylglutaryl coenzyme A (HMG-CoA) reductase activity through a post-transcriptional mechanism, which induces degradation of the reductase enzyme [19]. An unsaturated side-chain is essential for inhibition of hepatic HMG-CoA reductase activity. On the other hand, tocopherols (vitamin E) are well known for their characteristic antioxidant activity, but they do not increase reductase degradation or lower serum total or LDL-cholesterol levels [10,16]. The positive effects of tocotrienols as hypocholesterolemic, antioxidant, and anticancer agents have been confirmed in animal systems and various cell lines by many investigators [15-22].

Moreover, the far superior efficacy of tocotrienols versus tocopherols (vitamin E) as antioxidants has been established, and δ-tocotrienol is found to be the most potent among the known tocotrienols [10,17,18,22]. Tocotrienols also show non-antioxidant properties in various *in vitro *and *in vivo *models. Perhaps most importantly, tocotrienols interact with the mevalonate pathway leading to the lowering of cholesterol levels, the prevention of cell adhesion to endothelial cells, the suppression of tumor cell growth, and glutamate-induced neurotoxicity [10-12,23,24].

The present investigation was carried out to evaluate the mechanisms by which tocotrienols inhibit inflammation. Murine systems were chosen in the present study, because they are relatively resistant to the lethal effects of endotoxin compared to rabbit and sheep [1,3,25]. Recently, rodent models have been developed which are very sensitive to bacterial endotoxin and thus, female mice were chosen due to their increased sensitivity to LPS-induced inflammation. These experiments were designed to test our hypothesis that tocotrienol treatment modulates proteasomal activity, induces corticosteroids synthesis, and blocks LPS-induced signaling pathways that contribute to the inflammatory process.

## Materials and methods

### Reagents

Highly purified, deep rough chemotype LPS (Re LPS) from *E. coli *D31m4 was prepared as described by Qureshi et al. [26]. For tissue culture studies, Dulbecco's Modified Eagle Medium (DMEM), heat-inactivated low-endotoxin fetal bovine serum (FBS), and gentamicin were all purchased from Cambrex (Walkersville, MD). Thioglycollate was purchased from Sigma, Aldrich (St. Louis, MO) and the RNeasy mini kit from QIAGEN sciences (Germantown, MD). Substrates for the chymotrypsin-like activity of the proteasome were purchased from Calbiochem (La Jolla, CA). Tocotrienol rich fractions (TRF) of palm oil were provided by Malaysian Palm Oil Board, Kuala Lumpur, Malaysia (previously known as "Palm Oil Research Institute of Malaysia" [PORIM], Kuala Lumpur, Malaysia). "Proteasome-Glo" assays kits for chymotrypsin-like activity (substrate: Suc LLVY-Glo, Succinyl-leucine-leucine-valine-tyrosine-aminoluciferin), trypsin-like activity (Z-LRR aminoluciferin, Z-leucine-arginine-arginine-aminoluciferin), and post-glutamase activity (post-acidic, substrate, ZnLPnLD-Glo, Z-norleucine-proline-norleucine-aspartate-aminoluciferin) of the proteasome were purchased from Promega (Madison, WI). RAW 264.7 cells (TIB 71) were purchased from American Type Culture Collection (Manassas, VA), and 4-week-old BALB/c female mice were obtained from The Jackson Laboratory (Bar Harbor, ME).

### Purification of α-tocopherol, α-, γ-, and δ-tocotrienols from TRF of palm oil

The individual components of tocotrienol rich fraction (TRF) of palm oil were purified as described recently [10]. The purity of α-tocopherol and individual tocotrienols were established by high pressure liquid chromatography (HPLC) against their respective pure standards [10].

### Effects of various tocols (tocopherols + tocotrienols) on the chymotrypsin-like activity of 20 S rabbit muscle proteasomes

Proteasomal activities of the 20 S rabbit muscle proteasomes (0.4 μg/mL) were assayed with synthetic peptide substrates in 0.02 M Tris-HCl buffer (pH 7.2). The substrate used for the chymotrypsin-like activity was 100 μM of succinyl-Leu-Leu-Val-Tyr-amino-methyl-coumarin. Fluorescence was measured (absorption at 360 nm and emission at 460 nm) using an FLX 800 microplate fluorescence reader (Bio-Tek Instruments, Winooski, VT).

### Cell culture and maintenance

The RAW 264.7 cells or mouse peritoneal macrophages were maintained in DMEM supplemented with 10% heat inactivated fetal bovine serum (FBS) and 10 mg gentamicin (in 500 mL) at 37°C in a humidified atmosphere with 5% CO_2 _as described previously [27]. Cells were cultured in 6-well plates as described in the legends to the figures.

### Effects of δ-tocotrienol (concentrations of 10 - 640 μM) after 60 min treatment on different proteasomal activities (chymotrypsin-like, trypsin-like, and postglutamase) in RAW 264.7 whole cells

In order to check the comparative inhibitory effect of tocols, only the most potent δ-tocotrienol was tested on the chymotrypsin-like, trypsin-like, and post-glutamase activities of the proteasome using RAW 264.7 whole cells; the following experiment was carried out. RAW 264.7 cells (10 × 10^4 ^cells/100 μl/well) were added in white plates 96-well, Fisher, 0877126), followed by the addition of various concentrations of δ-tocotrienol (10, 20, 40, 80, 160, 320, or 640 μM in 100 μL; dissolved in 0.2% dimethyl sulfoxide (DMSO). The mixtures were incubated at 37°C in an incubator at 5% CO_2 _for 60 min. After incubation period, the cells in the 96-well plates were taken out 20 min prior to the addition of Caspase-Glo reagent (brought to room temperature before addition to the wells). Caspase-Glo reagent (100 μL) was added to each well to a total volume of 200 μL/well (tris buffer, pH 7.5; 0.02 M). The plate were covered with a plate sealer, removed from light and incubated at room temp for 10 min. The relative luminescence units (RLU) of assays were read with a Promega Plate Luminometer. The chymotrypsin-like, trypsin-like, or post-glutamase activities were quantitated by measuring luminescence after stimulation of RAW 264.7 whole cells with various doses of δ-tocotrienol in a Luminometer (Promega), according to the directions of manufacturer.

### Effects of various tocols (α-tocopherol, α-tocotrienol, γ-tocotrienol or δ-tocotrienol) on the secretion of TNF-α in LPS-stimulated RAW 264.7 cells

The levels of TNF-α in RAW 264.7 cell culture supernatants were determined after treatment with LPS (1 ng/mL) and various doses (4, 8, 16 μM) of α-tocopherol, α-tocotrienol, γ-tocotrienol or δ-tocotrienol by Quantikine M ELISA kit (R&D System, Minneapolis, MN) according to manufacturer's instructions. The lower limit of detection for TNF-α in this method is approximately, 5.0 pg/mL [28,29]. The TNF-α levels in mouse's serum or thioglycollate-elicited peritoneal macrophages were also quantified by using the same method [28,29].

### Effects of α-, γ-, and δ-tocotrienols on release of LPS-induced TNF-α in serum of 6-wk-old female BALB/c mice

All mice used in this study received humane care in compliance with the principles of laboratory animal care formulated by the National Society of Health Guide for the Care and Use of Laboratory Animals (DHHS Publication No. [NIH] 85 - 23, revised 1985). The experimental procedures involving animals were reviewed and approved by the Institutional Animal Care and Use Committee of UMKC, Medical School, MO. Female BALB/c mice were acclimatized to the new environment for fourteen days and were fedad libitum regular commercial mice diet and had free access to water throughout the experiment. A 12 h light and 12 h dark cycle was maintained during feeding period. All samples were dissolved in 5% triethylamine solution.

Seven groups of 4-wk-old female BALB/c mice (3/group) were acclimatized (for two wks), and then injected either with saline (two control groups), dexamethazone or various doses (2.5, 5.0, and 10.0 μg/kg body weight) of α-tocopherol, α-tocotrienol, γ-tocotrienol, or δ-tocotrienol. One h later, all mice were injected intraperitoneally with pure LPS (*E. coli *D31m4; 10 ng/mouse). After two h, all mice were sacrificed, serum was collected, and the levels of TNF-α were quantified using a radioimmunoassay kit according to the manufacturer's directions [28,29].

### Effects of α-, γ-, and δ-tocotrienols on the induction of corticosterone and adrenocorticotropic hormone (ACTH) in serum of LPS-stimulated 6-wk-old female BALB/c mice

To further probe the anti-inflammatory effects of various tocotrienols in mice, we queried whether tocotrienols would also induce the serum levels of corticosterone and adrenocorticotropic hormone (ACTH) after LPS treatment of mice as reported recently [3]. The levels of serum (used from the above experiment) corticosterone and ACTH were estimated according to published method [1,3].

### Isolation of thioglycollate-elicited peritoneal macrophages from BALB/c mice, total cellular RNA isolation and RT-PCR

Ten 4-wk-old female BALB/c mice were acclimatized to the new environment for fourteen days, and thioglycollate-elicited peritoneal macrophages were prepared as described previously [29]. Macrophages (2 × 10^5^) were treated with either pure LPS (10 ng/treatment), LPS + α-tocopherol (25, 50, or 100 μM), LPS + δ-tocotrienol (10, 20, or 40 μM). All the samples were dissolved in 0.2% dimethyl sulfoxide (DMSO). The assay mixtures were incubated at room temperature for 4 h and then centrifuged at 2,000 rpm for 20 min. The supernatants were removed and concentrations of TNF-α were determined using Quantikine M ELISA kit (R&D System, Minneapolis, MN) according to the manufacturer's directions [29]. The total RNA was isolated from each pellet with RNeasy mini kit according to the manufacturer's instructions. To check the purity of the total RNA, Reverse transcriptase polymerase chain reaction (RT-PCR) was conducted using a 1-step kit (Qiagen, Chatsworth, CA) according to the manufacturer's instructions.

### Detection of cell viability

Viability of peritoneal macrophages treated with and without LPS plus dexamethasone (positive control), α-tocopherol or various doses of δ-tocotrienol was determined by trypan blue dye exclusion or a quantitative colorimetric assay with 3-(4,5)-dimethylthiozol-2,5-diphenyltetrazolium bromide (MTT) as described previously [30].

### Statistical analyses

The analyses demonstrated the affect of various isomers of tocols (α-tocopherol or α-, γ-, δ-tocotrienols) within the groups. Stat View software (version 4.01, Abacus Concepts, Berkeley, CA) was used for the analyses of treatment-mediated effects as compared to the control group. Treatment-mediated differences in various inflammatory markers variables were evaluated using a two-way ANOVA, and when F test indicated a significant effect, the differences between the means were analyzed by a Fisher's protected least significant and least difference test. Data were reported as means ± SD in text and Tables. The level for statistical significance level was established at 5% (*P *< 0.05).

## Results

The results of the present study on the inhibition of inflammation by tocotrienols are presented in the same in two different formats. For each figure, 'A' shows the raw values for each of the treatments and controls, and 'B' shows the percent change compared to controls.

### Inhibition of chymotrypsin-like activity of 20 S rabbit muscle proteasomes by various tocols (tocopherols + tocotrienols)

**Figure 2:**
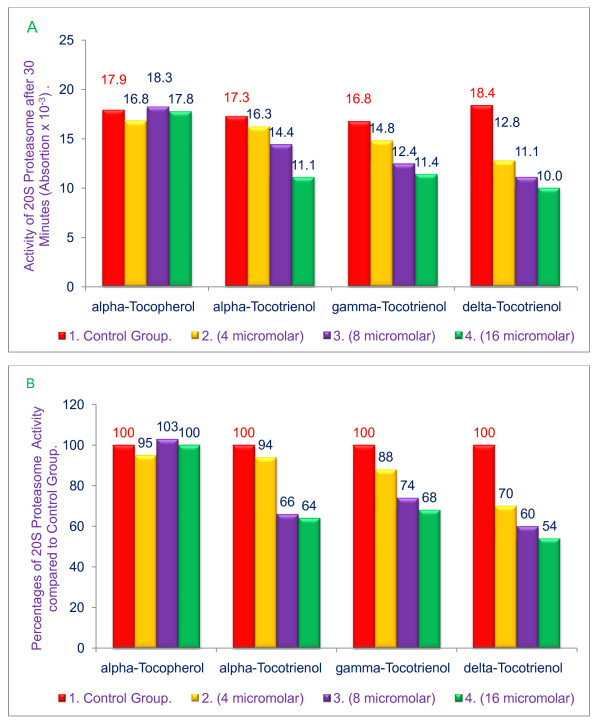
**Effects of various tocol treatments on the chymotrypsin-like activity of 20 S rabbit muscle proteasomes**. Chymotrypsin-like activity was carried out by using synthetic dipeptide substrate III (Suc-Leu-Leu-Val-Tyr-AMC) in buffer pH 7.5, 0.02 M. Rabbit muscle proteasomes were used. The compounds were dissolved in 2% dimethyl sulfoxide (DMSO). Fluorescence absorption was measured at excitation = 360 nm and emission = 460 nm, A = values; B = percentages.

We have previously shown that the proteasome is a central regulator of inflammation; we therefore queried whether tocotrienols also affect proteasome activity, thus affecting the induction of cytokines. The results of this study reveals that inhibition of chymotrypsin-like activity of the 20 S rabbit muscle proteasomes was dose-dependent between 4 μM and 16 μM for α-tocotrienol (6% - 36%), γ-tocotrienol (12% - 32%), and δ-tocotrienol (51% - 55%) as compared to control groups (Figure 2A &2B). An insignificant reduction (5%) in 20 S proteasomes activity was observed with α-tocopherol (Figure 2A &2B). These studies further suggest that δ-tocotrienol is the most effective isomer of the tocotrienols for inhibiting the chymotrypsin-like activity of rabbit muscle proteasomes.

### Impact of δ-tocotrienol (concentrations of 10 - 640 μM) on different proteasomal active sites (chymotrypsin-like, trypsin-like, and post-glutamase) in RAW 264.7 whole cells after 60 min treatment

**Figure 3:**
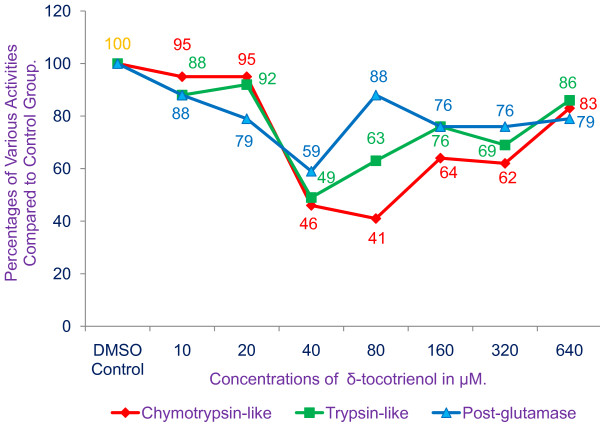
**Effects of δ-tocotrienol (concentration 10 μM - 640 μM) on proteasomal active sites, chymotrypsin-like, trypsin-like and post-glutamase, after 60 min treatment of RAW 264.7 whole cells**. Proteasome-Glo (Promega) chymotrypsin-like, trypsin-like, and post-glutamase cell based assays were carried out. 10,000 cells were plated per well in 100 μL of media in a 96-well white colored plate. Cells were allowed to adhere to plates for 2 h prior to testing. At the start of the test, media or 0.4% DMSO + media (used as control = CD), or δ-tocotrienol (concentrations 10 μM - 640 μM) dissolved in 0.4% DMSO were added to each well for a duration of 1 h. The mixtures in the plates were assayed according to Promega Protocol. Plates were read with a "Promega Luminometer" according to the directions of manufacturer, which give relative luminescence units (RLV) values.

The effects of δ-tocotrienol on the chymotrypsin-like, trypsin-like, and post-glutamase activities of proteasomes were also carried out in RAW 264.7 murine macrophages. The chymotrypsin-like activities decreased to 46% of controls with 40 μM δ-tocotrienol treatment of cells and then increased gradually as shown in Figure 3. A similar trend was observed with the trypsin-like activity and post-glutamase activities in response to δ-tocotrienol (Figure 3).

### Inhibition of the secretion of TNF-α in LPS-stimulated RAW 264.7 cells by various tocols (α-tocopherol, α-tocotrienol, γ-tocotrienol or δ-tocotrienol)

**Figure 4:**
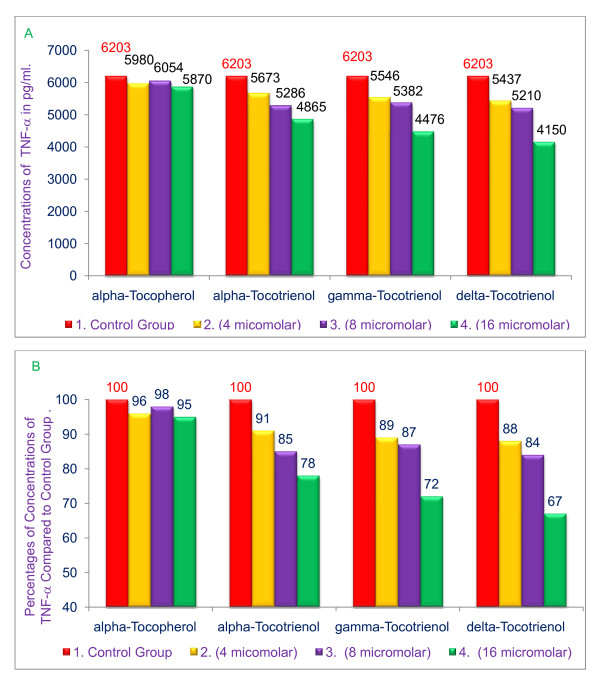
**Effects of various tocols on secretion of TNF-α in LPS-stimulated RAW 264**. 7 cells. RAW 264.7 cells (500 μL) were adhered in wells for 2 h at room temperature. The cells were then treated with various concentrations of α-tocopherol, or α-, γ-, or δ-tocotrienols (100 μL) for 1 h, then the wells were treated with LPS (1 ng/well; 400 μL) for 4 h. The supernatants were then transferred in glass vials and stored at -20°C for subsequent TNF-α assays. Cell viability was determined for all treatment and control groups. The experimental solution were prepared by dissolving highest concentration amount of α-tocopherol or α-, γ-, δ-tocotrienols in 1.0 mL DMSO = X. Solution × (50 μL) was mixed with 950 μL of media = Y. The remaining required concentrations were prepared with Y (1:2 dilutions). The TNF-α assays were carried out using an ELISA kit, and kit control value varies 266 - 444 (287), A = values; B = percentages.

The effects of tocotrienols on the secretion of TNF-α with, and without, LPS-stimulation in RAW 264.7 cells was investigated. A significant reduction of secretion of TNF-α, 9% - 33% (*P *< 0.05) was observed with tocotrienols in LPS-stimulated RAW 264.7 cells in a dose-dependent manner (Figure 4A &4B). Tocotrienols did not inhibit TNF-α secretion in the absence of LPS stimulation (data not shown in the figure). Cell viability for all above experiments was 90% - 95%.

### α-, γ-, and δ-Tocotrienols reduced serum TNF-α levels in LPS-stimulated 6-wk-old BALB/c mice

**Figure 5:**
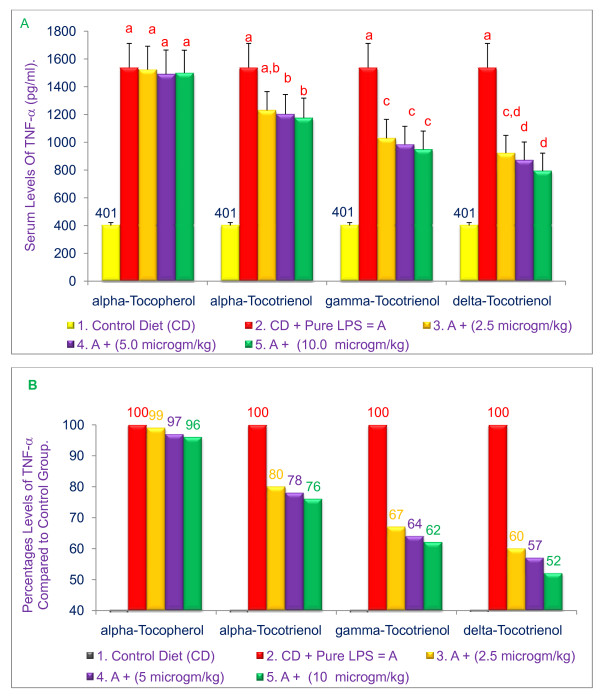
**Effect of various tocols on serum TNF-α levels in LPS-stimulated, 6-week-old female BALB/c mice**. The BALB/c female mice were acclimatized for 2 wk. Three mice for each concentration were injected intraperitoneally (i.p.) with compounds suspended in 5% triethylamine solution (0.2 mL/mouse) one h before LPS challenge. Mice were bled 2 h later to collect serum. The serum samples were stored at -20°C to for subsequent TNF-α analysis by ELISA. Values in a column with a different superscript letter are significantly different at *P *< 0.05, A = values; B = percentages.

To further probe the anti-inflammatory effects of various tocols in mice, we queried whether dietary supplementation with tocotrienols would also block LPS-induced TNF-α secretion in mice. A dose-dependent inhibition (20% to 48%; *P *< 0.05) of LPS-induced serum TNF-α levels was observed with α-, γ-, and δ-tocotrienols as compared to control diets (Figure 5A &5B). The reduction with α-tocopherol (5% - 9%) was insignificant as compared to control or tocotrienol treatments (Figure 5A &5B). These results also suggest that δ-tocotrienol is the most effective isomer in reducing LPS-induced TNF-α secretion in mice.

### α-, γ-, and δ-Tocotrienols induce corticosterone in serum of 6-wk-old BALB/c mice

**Figure 6:**
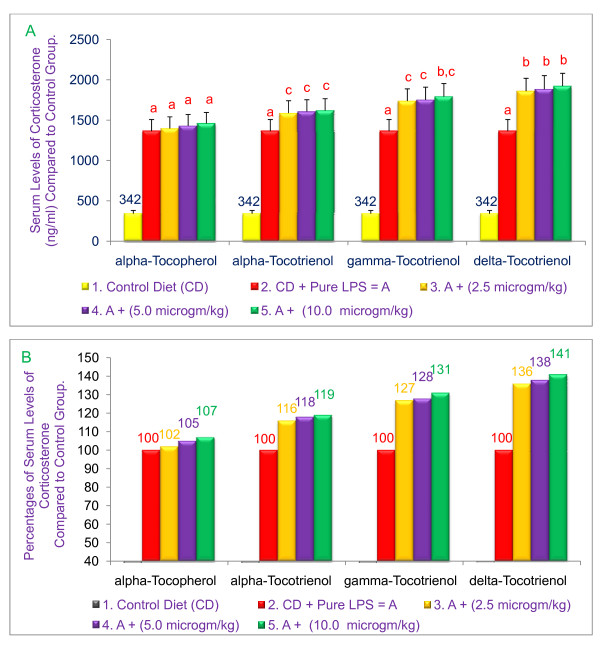
**Effects of α-tocopherol, α-tocotrienol, γ-tocotrienol and δ-tocotrienol on the induction of corticosterone in serum of LPS-stimulated 6-wk-old BALB/c female mice**. Three mice for each concentration were injected i.p. with tocols in 5% triethylamine solution (0.2 mL/mouse) one h prior to LPS challenge, after conditioning them for 14 days. Mice were bled after 2 h later to collect serum. Serum corticosterone levels were determined according to a published procedure (3). Values in a column with a different superscript letters are significantly different at *P *< 0.05, A = values; B = percentages.

We have previously shown that LPS treatment of mice induces high endogenous corticosterone levels, and that corticosterone levels are inversely correlated with TNF-α serum concentrations [9]. Therefore, to further explore mechanisms responsible for the anti-inflammatory effects of tocotrienols, we queried whether tocols increase corticosterone levels produced in response to LPS. For this experiment BALB/c mice were injected i.p. with tocotrienols. A significant (*P *< 0.02) rise in LPS-induced serum corticosterone (19%, 31%, and 41%) levels was induced by treatment with α-, γ-, or δ-tocotrienols as compared to the control group (Figure 6A &6B). These results suggest that the decrease in the synthesis of TNF-α could be due to the rise in the endogenous corticosteroids, which may modulate the synthesis of inflammatory cytokines [3].

### α-, γ-, and δ-Tocotrienols induce adrenocorticotropic hormone in serum of 6-wk-old BALB/c mice

**Figure 7:**
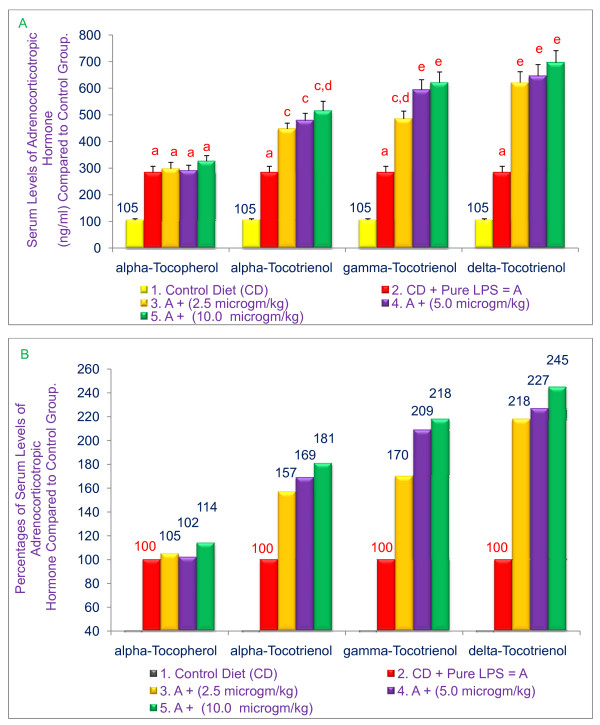
**Effects of α-tocopherol, α-tocotrienol, γ-tocotrienol and δ-tocotrienol on the induction of adrenocorticotropic hormone in serum of LPS-stimulated 6-wk-old BALB/c female mice**. Three mice for each concentration were injected i. p. with various tocols in 5% triethylamine solution (0.2 mL/mouse) one hr prior to LPS challenge, after conditioning them for 14 days. Mice were bled after 2 h later to collect serum. Serum adrenocorticotropic hormone levels were determined according to published procedure (3). Values in a column with a different superscript letters are significantly different at *P *< 0.05, A = values; B = percentages.

The impact of α-tocopherol and various tocotrienols on the levels of adrenocorticotropic hormone was also determined in the serum obtained from the experiment described above. A corresponding significant (*P *< 0.02) dose-dependent rise in LPS-induced serum adrenocorticotropic hormone levels was observed (81%, 118%, and 145%) by treatment with α-, γ-, or δ-tocotrienols, respectively, as compared with the control group (Figure 7A &7B). An insignificant rise in the levels of corticosterone (7%) and adrenocorticotropic hormone (14%) with α-tocopherol was also observed (Figure 6B &7B). In summary, these results clearly indicate that δ-tocotrienol is most effective in inhibiting the serum levels of TNF-α and also in inducing the serum levels of corticosterone and adrenocorticotropic hormone as compared to α-, γ-tocotrienols and α-tocopherol, which were the least effective compounds as compared to their respective control groups.

### δ-Tocotrienol inhibits LPS-induced TNF-α, IL-1β, IL-6, and iNOS gene expression in thioglycollate-elicited peritoneal macrophages of 6-wk-old BALB/c mice

**Figure 8:**
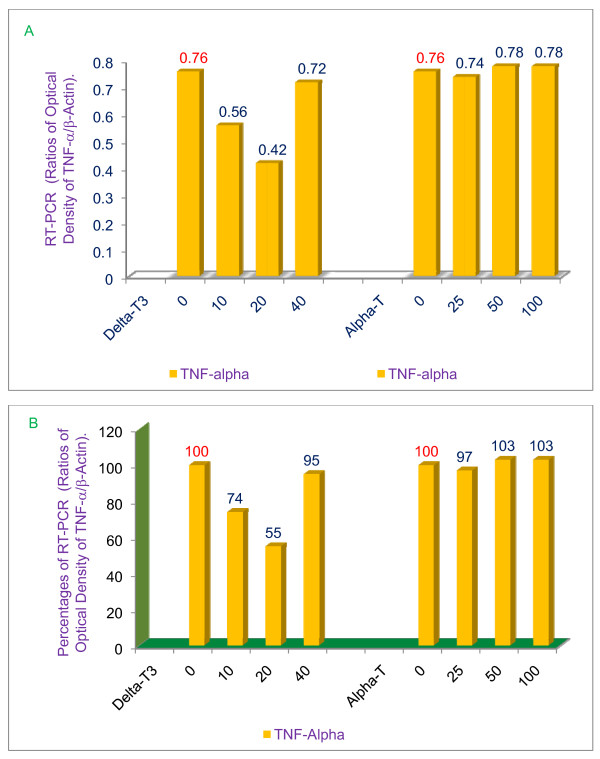
**Effects of α-tocopherol and δ-tocotrienol on the gene expression of TNF-α in LPS-stimulated peritoneal macrophages of 6-week-old BALB/c female mice**. The RT-PCR procedure is described in the experimental section. The ratios of relative optical density (TNF-α/β-actin) of each treatment for TNF-α was used to draw this figure, A = values; B = percentages.

**Figure 9:**
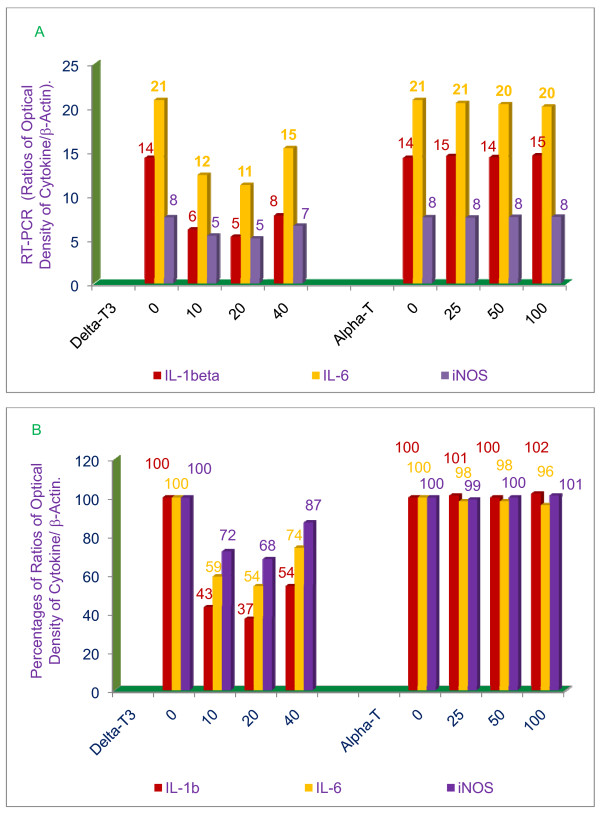
**Effects of α-tocopherol and δ-tocotrienol on the gene expression of IL-1β, IL-6, and iNOS in LPS-stimulated peritoneal macrophages of 6-week-old BALB/c female mice**. The RT-PCR procedure is described in the experimental section. The ratios of relative optical density (cytokines/β-actin) of each treatment for each marker was used to draw this figure, A = values; B = percentages.

In order to define the mechanism of action of tocotrienols with respect to lowering inflammation, thioglycollate-elicited peritoneal macrophages were prepared from BALB/c female mice, and treated with α-tocopherol, or δ-tocotrienol 1 h before, or at the same time as LPS. After 4 h of the treatment, total cellular RNA was extracted and reverse-transcribed, and gene analysis was performed by RT-PCR and southern blot analyses. The results of gene expression studies clearly demonstrate that δ-tocotrienol is most effective in blocking LPS-induced gene expression of TNF-α Figure 8A &8B, IL-1β, IL-6 and iNOS at low doses of 10 μM and 20 μM (Figure 9A &9B).

There were significant (*P *< 0.02) reductions of TNF-α (45%), IL-1β (63%), IL-6 (46%) and iNOS (32%) mRNA with a dose of 20 μM compared to their respective control values, and significant increases (40%, 17%, 26%, 13%) in the levels of these mRNA's, with a dose of 40 μM of δ-tocotrienol, as compared to their respective 20 μM control values (Figure 8B &9B). These results are in agreement with earlier results of tocotrienols on the activities of chymotrypsin-like, trypsin-like and post-glutamase in RAW 264.7 whole cells (Figure 3). The α-tocopherol (doses of 25, 50 or 100 μM) did not have any impact on the gene expression of TNF-α, IL-1β, IL-6 and iNOS compared to their respective control values (Figure 8A, Figure 9B).

## Discussion

It is well established that LPS-induced TNF-α production requires activation of NF-κB and activation of proteasomal activity. The results presented here demonstrate that tocotrienols block production of key pro-inflammatory cytokines induced by LPS. We hypothesized that this blockade of LPS-induced inflammation by tocotrienols could be due to modulation of proteasomal activity [31], since tocotrienols have been shown to be effective inhibitors of β-hydroxy-β-methylglutaryl coenzyme A (HMG-CoA) reductase, (the rate-limiting enzyme in cholesterol biosynthesis), and are very potent antioxidant and hypocholesterolemic agents [12]. It has also been demonstrated that HMG-CoA reductase is degraded via the ubiquitin-proteasome pathway [32,33]. We have shown that tocotrienols can inhibit chymotrypsin-like activity of 20 S rabbit muscle proteasome, and that δ-tocotrienol specifically inhibits the chymotrypsin-like, trypsin-like and post-glutamase activities of the proteasome in a dose-dependent manner in RAW 264.7 whole cells at concentrations below 80 μM; at higher concentrations (80 - 640 μM) tocotrienole enhance these activities as has been previously reported for lactacystin [34,35].

Tocotrienols also inhibit the LPS-stimulated secretion of TNF-α in RAW 264.7 cells and in serum of BALB/c mice, and a corresponding significant (*P *< 0.02) rise was observed in the serum levels of corticosterone and adrenocorticotropic hormone compared to controls. Moreover, δ-Tocotrienol is effective in reducing LPS-induced gene expression of TNF-α, IL-1β IL-6 and iNOS at low concentrations. Interestingly, at high concentrations δ-Tocotrienol activates chymotrypsin-like, trypsin-like, and post-glutamase activities, and upregulates LPS-induced gene expression of TNF-α, IL-1β, IL-6 and iNOS. It seems that tocotrienols actually upregulates the proteasomal activity, and thus can upregulate LPS-stimulated transcription of several pro-inflammatory genes at higher concentrations. Therefore, the capacity of δ-tocotrienol to modulate inflammation may be attributable, in part, to inhibition and activation of the chymotrypsin-like, trypsin-like and post-glutamase' activities of mouse macrophages.

The present results also demonstrated that δ-tocotrienol is the most effective tocol among the known, α- and γ-tocotrienols for inhibition or induction of various cytokines involved in inflammation, whereas α-tocopherol does not play any role in inhibiting LPS-induced inflammation. This conclusion has been further confirmed in a recent study [32] which demonstrated that δ-tocotrienol is very effective in inducing ubiquitination and degradation of HMG-CoA reductase. δ-tocotrienol also blocks the processing of sterol regulatory element-binding proteins-2 (SREBPs-2) which, when activated, can enhance transcription of genes encoding cholesterol biosynthetic enzymes, including HMG-CoA reductase [32]. On the other hand, tocopherols neither increase degradation of reductase, nor decrease SREBP-2 processing [32].

The unsaturated isoprenoid side-chain in tocotrienol molecules is important for its biological activity and it was suggested that this could stimulate binding of HMG-CoA reductase to Insig (endoplasmic reticulum membrane protein). Alternatively, the side-chain might allow improved penetration and distribution in cell membranes [32,33]. This may contribute to the differences observed in the potency of tocotrienols versus tocopherols in stimulating HMG-CoA reductase *in vitro *ubiquitination [32,33] thus confirming our previous and present results showing that δ-tocotrienol (one methyl group) is more potent than γ-tocotrienol (two methyl groups) due to the number of methyl groups which abolishes regulatory activity with respect to HMG-CoA reductase degradation and SREBP-2 processing [10,11,32].

## Conclusions

Although LPS is a potent inducer of inflammation via the proteasome, it also activates an anti-inflammatory response by inducing corticosteroid production. In summary, δ-tocotrienol treatment has several anti-inflammatory effects that could be mechanistically analogous to the modulation of proteasomal activity by lactacystin, a well established proteasome inhibitor that can either increase or decrease proteasomal activity under different conditions [34,35]. The results of our current study also demonstrate that tocotrienols increase levels of adrenocorticotropic hormone (ACTH) and corticosteroids produced in response to LPS. The mechanisms by which LPS plus tocotrienols stimulate the hypothalamus-pituitary-adrenal (HPA) axis and the sites of action are currently unclear [3]. Regardless of the mechanism, however, the capacity of tocotrienols to increase the host corticosteroid response to LPS is likely to modulate inflammation. Other possible mechanisms for the anti-inflammatory properties of tocotrienols also include its superior antioxidant activity compared to α-tocopherol (vitamin E, [10,17,22]). All these latter mechanisms such as corticosteroid induction and antioxidant activities may be dependent on the proteasomal modulation by tocotrienols.

## Abbreviations

ACTH: adrenocorticotropic hormone; DMSO: dimethyl sulfoxide; FBS: fetal bovine serum; IL-1β: interleukin-1β; IL-6: interleukin-6; IL-8: interleukin-8; HMG-CoA: β-hydroxy- β-methylglutaryl coenzyme A; HPLC: high pressure liquid chromatography; iNOS: inducible nitric oxide synthase; LDL: low density lipoprotein; LPS: lipopolysaccharide; MTT: 3-(4,5)-dimethyl-2,5-diphenyltetrazolium bromide; RNA: ribonucleic acid; RT-PCR: reverse-phase transcriptase polymerase chain reaction; SREBP-2: sterol regulatory element-binding protein-2.; TCL: total cholesterol; Tocols: mixtures of α-, β-, γ-, δ-tocopherols + α-, β-, γ-, δ-tocotrienols; TNF-α: tumor necrosis factor-α; TRF: tocotrienol rich fraction.

## Competing interests

The authors declare that they have no competing interests.

## Authors' contributions

JCR (graduate student) has carried out most of the experiments. All the authors were involved in the design of the study. CJP edited the manuscript. All the authors have read and approved the final version.
